# Syk Kinase-Coupled C-type Lectin Receptors Engage Protein Kinase C-δ to Elicit Card9 Adaptor-Mediated Innate Immunity

**DOI:** 10.1016/j.immuni.2011.11.015

**Published:** 2012-01-27

**Authors:** Dominikus Strasser, Konstantin Neumann, Hanna Bergmann, Mohlopheni J. Marakalala, Reto Guler, Anna Rojowska, Karl-Peter Hopfner, Frank Brombacher, Henning Urlaub, Gottfried Baier, Gordon D. Brown, Michael Leitges, Jürgen Ruland

**Affiliations:** 1 Institut für Klinische Chemie und Pathobiochemie, Klinikum rechts der Isar, Technische Universität München, 81675 Munich, Germany; 2 Laboratory of Signaling in the Immune System, Helmholtz Zentrum München - German Research Center for Environmental Health, 85764 Neuherberg, Germany; 3 Division of Immunology, Institute of Infectious Disease and Molecular Medicine, Faculty of Health Sciences, University of Cape Town, Anzio Road, Observatory 7925, Cape Town, South Africa; 4 International Center for Genetic Engineering and Biotechnology, University of Cape Town, Anzio Road, Observatory 7925, Cape Town, South Africa; 5 Gene Center and Department of Biochemistry, Ludwig-Maximilians-Universität München, 81377 Munich, Germany; 6 Bioanalytical Mass Spectrometry Group, Max-Planck-Institut für biophysikalische Chemie, 37077 Göttingen, Germany; 7 Bioanalytics, Department of Clinical Chemistry, University Medical Center Göttingen, 37075 Göttingen, Germany; 8 Department for Medical Genetics, Molecular & Clinical Pharmacology, Innsbruck Medical University, 6020 Innsbruck, Austria; 9 Aberdeen Fungal Group, Section of Immunology and Infection, Institute of Medical Sciences, University of Aberdeen, AB25 2ZD, UK; 10 The Biotechnology Centre of Oslo, University of Oslo, P.O. Box 1125, Blindern, 0317 Oslo, Norway

## Abstract

C-type lectin receptors (CLRs) that couple with the kinase Syk are major pattern recognition receptors for the activation of innate immunity and host defense. CLRs recognize fungi and other forms of microbial or sterile danger, and they induce inflammatory responses through the adaptor protein Card9. The mechanisms relaying CLR proximal signals to the core Card9 module are unknown. Here we demonstrated that protein kinase C-δ (PKCδ) was activated upon Dectin-1-Syk signaling, mediated phosphorylation of Card9 at Thr231, and was responsible for Card9-Bcl10 complex assembly and canonical NF-κB control. *Prkcd*^−/−^ dendritic cells, but not those lacking PKCα, PKCβ, or PKCθ, were defective in innate responses to Dectin-1, Dectin-2, or Mincle stimulation. Moreover, *Candida albicans-*induced cytokine production was blocked in *Prkcd*^−/−^ cells, and *Prkcd*^−/−^ mice were highly susceptible to fungal infection. Thus, PKCδ is an essential link between Syk activation and Card9 signaling for CLR-mediated innate immunity and host protection.

## Highlights

► Dectin-1-Syk stimulation activates PKCδ in dendritic cells ► PKCδ controls Card9-Bcl10 complex assembly for canonical NF-κB activation ► *Prkcd*^−/−^ cells are defective in Dectin-1-, Dectin-2-, or Mincle-triggered responses ► PKCδ is essential for innate antifungal immunity and host protection in vivo

## Introduction

The innate immune system uses germline-encoded pattern recognition receptors (PRRs) to sense microbial pathogens or other forms of danger, leading to the induction of inflammatory responses and host defense (Medzhitov, 2009). Bona fide PRRs with signaling capacities that reprogram gene expression include Toll-like receptors (TLRs), nucleotide-oligomerization domain (Nod)-like receptors (NLRs), retinoic acid-inducible gene-I (RIG-I)-like helicases (RLRs), and the recently emerging family of spleen tyrosine kinase (Syk)-coupled C-type lectin receptors (CLRs) (Kerrigan and Brown, 2011; Osorio and Reis e Sousa, 2011). Central to the ability of these PRRs to elicit inflammation and innate immunity is their capacity to activate the canonical NF-κB signaling cascade (Vallabhapurapu and Karin, 2009).

Dectin-1, the first Syk-coupled CLR found to be crucial for mammalian host protection, still serves as a paradigm for CLR signaling (Brown and Gordon, 2001; Mócsai et al., 2010; Taylor et al., 2007). This receptor recognizes β-glucan carbohydrates in the cell walls of fungi. Genetic studies in humans and mice have revealed essential functions for Dectin-1 in antifungal host protection (Ferwerda et al., 2009; Taylor et al., 2007). To induce signaling, Dectin-1 contains an immunoreceptor tyrosine-based activation motif (ITAM)-like motif in its intracellular tail, which is responsible for Syk engagement (Kerrigan and Brown, 2011; Mócsai et al., 2010). Additional Syk-coupled CLRs with critical roles in antifungal immunity are Dectin-2 and Mincle (Robinson et al., 2009; Saijo et al., 2010; Sato et al., 2006; Wells et al., 2008), which associate with the ITAM-containing adaptor molecule Fc receptor γ chain (FcRγ) (Mócsai et al., 2010; Sato et al., 2006; Wells et al., 2008). Notably, ITAM-Syk-coupled CLRs are not only involved in antifungal defense but are also implicated in innate immunity to mycobacteria (Ishikawa et al., 2009; Schoenen et al., 2010) or helminths (Ritter et al., 2010), as well as in inflammatory responses triggered by viruses or self-ligands (Chen et al., 2008; Kerrigan and Brown, 2011; Osorio and Reis e Sousa, 2011; Sancho et al., 2009; Yamasaki et al., 2008).

CLR signaling is initiated by receptor proximal Src family kinases that phosphorylate ITAMs or ITAM-like structures, resulting in Syk recruitment and activation (Kerrigan and Brown, 2011; Mócsai et al., 2010; Osorio and Reis e Sousa, 2011). Syk is then responsible for tyrosine phosphorylation of signaling intermediates that trigger downstream pathways, including mitogen-activated protein kinase (MAPK) and NF-κB signaling (Mócsai et al., 2010). A general mediator of CLR function is the adaptor protein Card9, which controls canonical IκB-kinase (IKK) and NF-κB activation (Gross et al., 2006; Hara et al., 2007; Mócsai et al., 2010) and which is required for inflammatory responses induced by Dectin-1 (Gross et al., 2006; Hara et al., 2007), Dectin-2 (Robinson et al., 2009), and Mincle (Schoenen et al., 2010; Werninghaus et al., 2009; Yamasaki et al., 2008). Consistent with the essential roles of the Card9 pathway in host defense, mice or humans with a genetic loss of Card9 function are immunodeficient and highly susceptible to fungal infections (Glocker et al., 2009; Gross et al., 2006). Still, even though the Syk-Card9 pathway is nonredundant in innate immunity, the molecular mechanisms that couple CLR proximal events to Card9 activation are undefined.

Here we used a proteomic approach to search for intracellular mediators of CLR-Syk function. With Dectin-1 signaling as a model, we report that PKCδ (encoded by *Prkcd*) is activated upon CLR stimulation and specifically required to induce Card9-Bcl10 complex assembly for TAK1 activation and canonical NF-κB control. We found that *Prkcd***^−/−^** dendritic cells are defective in innate immune responses to CLR stimulation and that *Prkcd***^−/−^** mice are highly susceptible to fungal infection. These results identify a fundamental mechanism for inflammatory cell activation, with essential roles in CLR-mediated innate immunity.

## Results

### Zymosan Stimulation Triggers Tyrosine Phosphorylation of PKCδ

**Figure 1:**
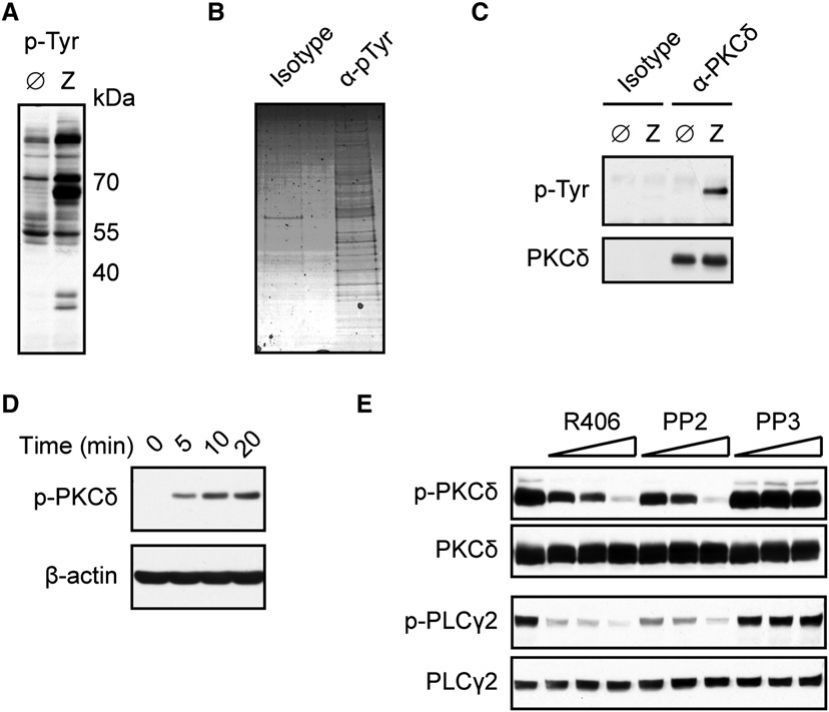
PKCδ Is Tyrosine Phosphorylated upon Dectin-1 Ligation (A) BMDCs were left untreated (Ø) or stimulated for 10 min with zymosan (Z). Cellular lysates were immunoblotted with phospho-Tyrosine (p-Tyr) antibodies. (B) BMDCs were stimulated with zymosan as in (A). Proteins were immunopurified with p-Tyr antibodies or IgG1 control (isotype) and visualized by Coomassie staining after electrophoresis. (C) BMDCs were left untreated or stimulated with zymosan as in (A). Proteins from lysates were immunoprecipitated with PKCδ antibodies or isotype control and analyzed by immunoblot with p-Tyr or PKCδ antibodies. (D) Immunoblot analysis of BMDCs stimulated with zymosan for various times and probed with antibodies against phospho-PKCδ (Tyr311) or β-actin. (E) BMDCs were left untreated or preincubated with Syk inhibitor R406 (0.5, 1, 2 μM), Src-kinase inhibitor PP2 (1.5, 3, 6 μM), or its inactive analog PP3 (1.5, 3, 6 μM) and stimulated for 10 min with zymosan. Lysates were analyzed by immunoblot with antibodies against phospho-PKCδ, PKCδ, phospho-PLCγ2, or PLCγ2. All results are representative of at least three independent experiments.

To gain insights into the mechanisms of Dectin-1-Syk signaling, we stimulated primary bone marrow-derived dendritic cells (BMDCs) from mice with zymosan. Zymosan is a fungal cell wall preparation composed primarily of β-glucans and functions as a strong Dectin-1 agonist. In addition, it contains ligands for Dectin-2 and TLR2 (Brown and Gordon, 2001; Gross et al., 2006; Robinson et al., 2009; Taylor et al., 2007). As expected, zymosan stimulation induced tyrosine phosphorylation of multiple target proteins (Figure 1A). To reveal the identity of these molecules in an unbiased manner, we affinity purified the tyrosine-phosphorylated proteins and analyzed these by mass spectrometry (MS) (Figure 1B). The 20 specifically tyrosine-phosphorylated proteins with the highest Mascot scores included four kinases: the receptor proximal Src family members Lyn and Fgr, together with Syk and PKCδ (data not shown). Because we were particularly interested in putative Syk effector molecules, we focused our attention on PKCδ.

To validate zymosan-induced PKCδ phosphorylation, we immunoprecipitated this specific kinase from stimulated BMDCs and subsequently performed immunoblots with phospho-tyrosine antibodies. Consistent with our MS data, zymosan treatment induced strong PKCδ tyrosine phosphorylation (Figure 1C). Then, we performed analyses with a phosphospecific antibody raised against PKCδ tyrosine (Tyr) 311, a residue involved in PKCδ activation (Konishi et al., 2001). Robust, activation-specific PKCδ Tyr311 phosphorylation was rapidly detected in response to zymosan treatment (Figure 1D). To test whether this PKCδ phosphorylation was mediated via Src and Syk signaling, we pharmacologically blocked these receptor proximal tyrosine kinases with the inhibitors PP2 or R406, respectively, before stimulation of the cells. Both inhibitors impeded zymosan-induced PKCδ Tyr311 phosphorylation (Figure 1E). Thus, we conclude from this first set of experiments that innate recognition of zymosan activates PKCδ through a Src and Syk tyrosine kinase-dependent mechanism.

### PKCδ Is Essential for CLR-Mediated Cytokine Production

**Figure 2:**
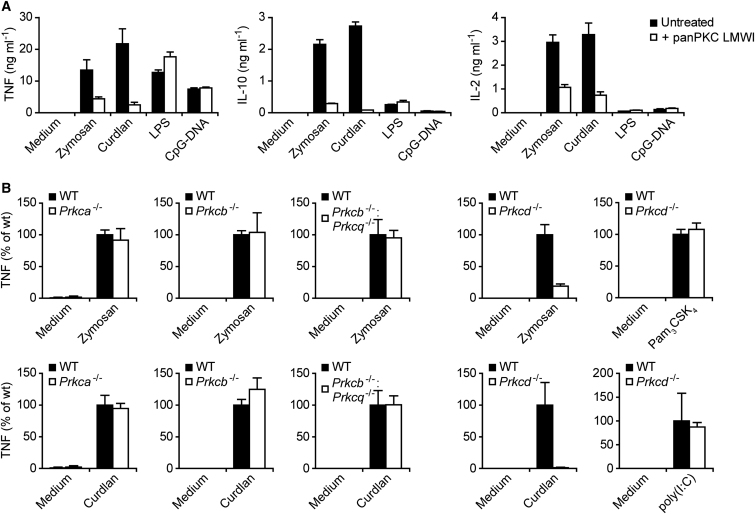
Selectively Impaired Dectin-1 Signaling in *Prkcd*^−/−^ BMDCs (A) BMDCs were left untreated or preincubated with PKC inhibitor panPKC LMWI (5 μM) and stimulated with zymosan (20 μg ml^−1^), curdlan (400 μg ml^−1^), LPS (200 ng ml^−1^), or CpG-DNA (2 μM) for 6 hr. TNF, IL-10, and IL-2 concentrations in the supernatants were assayed by ELISA. Data are expressed as means + SD of triplicate samples. (B) TNF production in *Prkca*^−/−^, *Prkcb*^−/−^, *Prkcq*^−/−^, or *Prkcd*^−/−^ BMDCs that were left untreated (Medium) or stimulated with zymosan (20 μg ml^−1^), curdlan (200 μg ml^−1^), Pam_3_CSK_4_ (30 ng ml^−1^), or poly(I:C) (30 μg ml^−1^) for 6 hr as indicated. Data are expressed as percent of WT + SD, derived from stimulations in triplicates.

Next, we were interested in the functional roles of PKCδ in Dectin-1-mediated innate immune responses. We therefore pretreated BMDCs with the selective PKC inhibitor panPKC LMWI (Hermann-Kleiter et al., 2006) prior to stimulation. panPKC LMWI inhibited TNF, IL-10, and IL-2 production in response to zymosan stimulation or upon specific Dectin-1 triggering with curdlan, a pure β-(1,3)-glucan polymer and selective agonist for Dectin-1 (Figure 2A; LeibundGut-Landmann et al., 2007). In contrast, treatment of the cells with panPKC LMWI did not inhibit cytokine secretion induced by TLR4 stimulation with LPS or TLR9 stimulation with CpG-DNA (Figure 2A).

**Figure 3:**
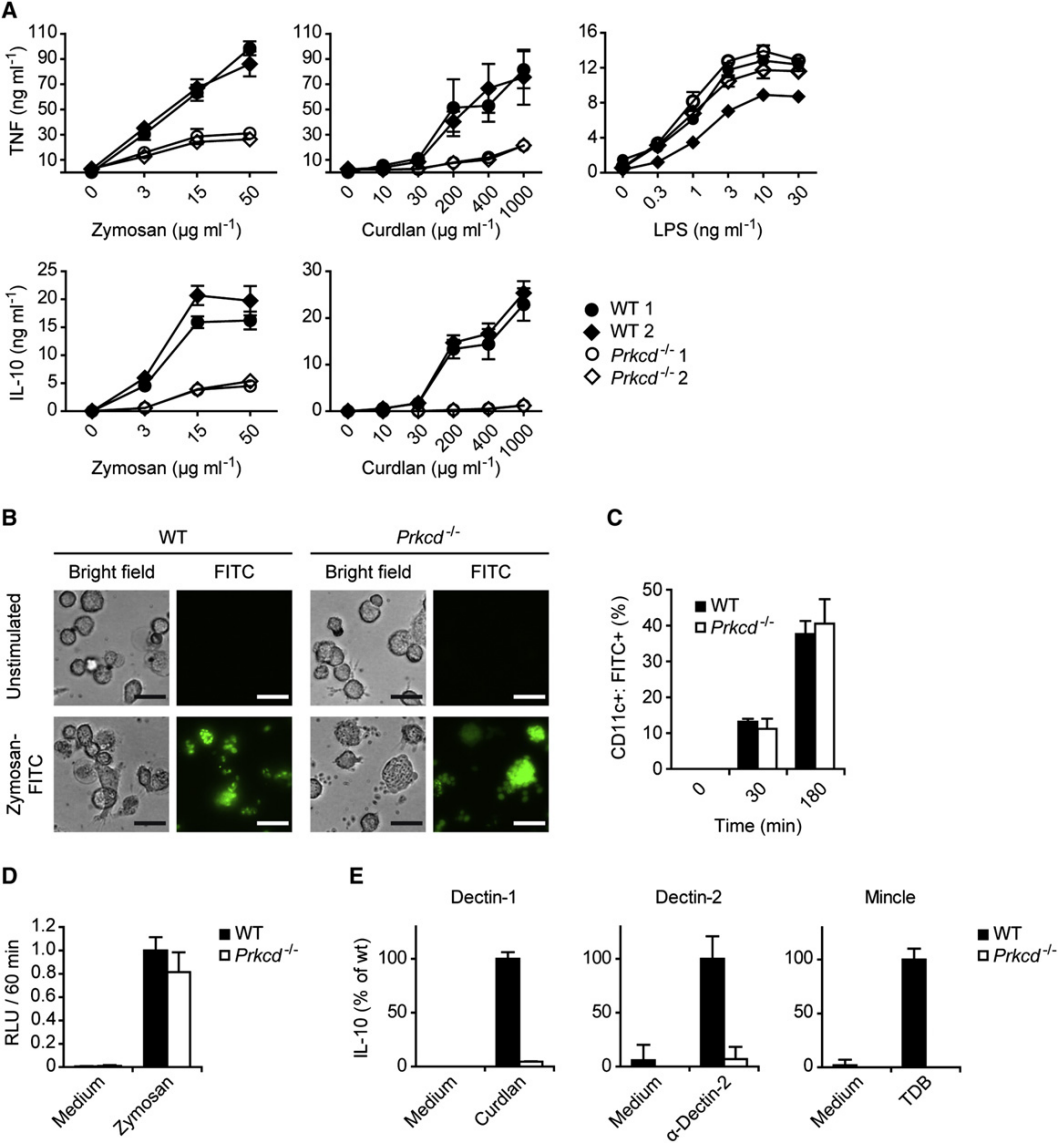
PKCδ Deficiency Particularly Impairs CLR Signaling but Not Phagocytosis (A) WT and *Prkcd*^−/−^ BMDCs were incubated with the indicated concentrations of zymosan, curdlan, or LPS. TNF and IL-10 concentrations in the supernatants were assayed by ELISA. Data are expressed as means ± SD of triplicates. (B) BMDCs from WT and *Prkcd*^−/−^ mice were incubated for 2 hr with FITC-zymosan particles (100 μg ml^−1^). FITC-zymosan internalization was visualized by fluorescence microscopy (scale bars represent 20 μm). (C) BMDCs were incubated with FITC-zymosan as in (B) for the times indicated. The frequencies of CD11c^+^ cells containing zymosan-FITC particles were quantified by FACS analysis. (D) BMDCs from WT and *Prkcd*^−/−^ mice were incubated with Luminol and were left untreated (Medium) or stimulated with zymosan. Luminescence was assayed for 60 min in 10 min intervals and relative light units (RLU) as a measure for ROS generation integrated over time. Results represent means + SD from three independent experiments. (E) WT and *Prkcd*^−/−^ BMDCs were stimulated through Dectin-1, Dectin-2, or Mincle with curdlan (20 μg ml^−1^), plate-bound Dectin-2 antibody, or TDB (100 μg ml^−1^), respectively. IL-10 concentrations in the cell culture supernatants were quantified by ELISA. Data are expressed as percent of WT + SD, derived from stimulations in triplicates. All results are representative of at least three independent experiments.

Because panPKC LMWI inhibits not only PKCδ but also other PKC isoforms (Hermann-Kleiter et al., 2006), we moved on to a genetic approach to dissect the functions of individual PKCs in CLR signaling. To this end, we generated BMDCs from mouse strains that are deficient in either PKCα (encoded by *Prkca*) (Leitges et al., 2002), PKCβ (encoded by *Prkcb*) (Leitges et al., 1996), PKCβ and PKCθ (encoded by *Prkcq*) (Pfeifhofer et al., 2003), or PKCδ (Leitges et al., 2001). Interestingly, BMDC differentiation was not impaired by the lack of any of these PKC isoforms (data not shown). Moreover, BMDCs deficient in PKCα, PKCβ, or PKCβ and PKCθ produced regular amounts of TNF and IL-10 in response to zymosan treatment or upon specific Dectin-1 stimulation with curdlan (Figure 2B and data not shown). In sharp contrast, *Prkcd*^−/−^ BMDCs were severely impaired in zymosan- or curdlan-induced cytokine production, although responses to TLR1-TLR2 stimulation with Pam_3_CSK_4_ or TLR3 triggering with long poly(I:C) were not reduced (Figure 2B). These findings indicate an essential and specific role for PKCδ in the Dectin-1 pathway. To characterize this function in more detail, we performed dose-response experiments with different PRR agonists. Again, we observed a critical requirement for PKCδ in zymosan- or curdlan-mediated TNF and IL-10 production, but cytokine production in response to TLR4 stimulation with LPS was not affected by the deletion of PKCδ (Figure 3A).

Because Dectin-1 signaling triggers not only the synthesis of cytokines but also the phagocytosis of fungal components, as well as the production of reactive oxygen species (ROS), we studied the role of PKCδ in these pathways. In contrast to cytokine production, the phagocytosis of zymosan particles was independent of PKCδ, as assessed by either fluorescence microscopy or flow cytometric quantification (Figures 3B and 3C). Moreover, the generation of ROS upon zymosan treatment was not significantly impaired in *Prkcd*^−/−^ BMDCs (Figure 3D).

Next we investigated whether PKCδ functions in Dectin-1-independent CLR responses by stimulating *Prkcd*^−/−^ cells with agonistic antibodies against Dectin-2 or with trehalose-6,6-dibehenate (TDB), a synthetic adjuvant analog of the mycobacterial cord factor that functions as a selective and specific agonist for Mincle (Schoenen et al., 2010). Similar to the Dectin-1 responses, both Dectin-2- and Mincle-induced IL-10 and TNF production were severely impaired in the absence of PKCδ (Figure 3E and data not shown). These findings indicate that PKCδ plays a general role in Syk-coupled CLR response pathways, where it is specifically required for the control of cytokine synthesis.

### PKCδ Regulates Dectin-1-Mediated NF-κB Signaling via Card9 Control

**Figure 4:**
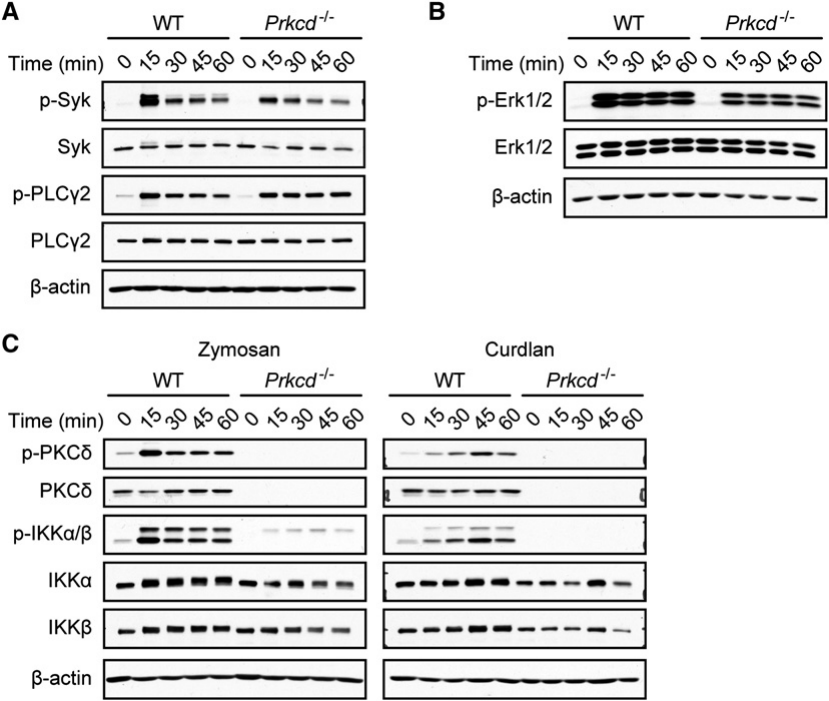
PKCδ Controls NF-κB Activation (A) Regular Syk and PLCγ2 activation in *Prkcd*^−/−^ cells. BMDCs from WT or *Prkcd*^−/−^ mice were stimulated with zymosan for the indicated times. Syk and PLCγ2 activation was determined by immunoblot with phospho-Syk or phospho-PLCγ2 antibodies. Immunoblotting with Syk, PLCγ2, and β-actin antibodies indicate equal protein loading. (B) WT or *Prkcd*^−/−^ BMDCs were stimulated with zymosan as indicated. Activation of the MAP kinases Erk1 and Erk2 was determined by immunoblot with phospho-Erk1/2 antibodies. Immunoblotting with Erk1/2 and β-actin antibodies indicates equal protein loading. (C) Defective NF-κB signaling in *Prkcd*^−/−^ BMDCs. Cells were stimulated with zymosan or curdlan as indicated. Lysates were analyzed by immunoblot with phospho-PKCδ, phospho IKKα/β and PKCδ, IKKα, IKKβ, and β-actin antibodies.

To define the molecular function of PKCδ in CLR signaling, we focused again on zymosan stimulation and selective Dectin-1 triggering (Figure 4). The overall tyrosine phosphorylation pattern, the activation of Syk, as well as the phosphorylation of the Dectin-1 signal transducer PLCγ2 (Xu et al., 2009) did not differ substantially between zymosan- or curdlan-stimulated WT and *Prkcd*^−/−^ cells (Figure 4A and data not shown). Moreover, PKCδ was to a large extent dispensable for Erk1 and Erk2 MAPK activation, as shown by the fact that Erk1 and Erk2 phosphorylations were only slightly reduced in *Prkcd*^−/−^ cells (Figure 4B). In sharp contrast, signaling to the canonical NF-κB pathway, a key driver of cytokine production, was almost completely blocked in zymosan-treated or Dectin-1-stimulated *Prkcd*^−/−^ BMDCs, as indicated by the lack of phosphorylation in the activation loops of IKKα and IKKβ (Figure 4C).

**Figure 5:**
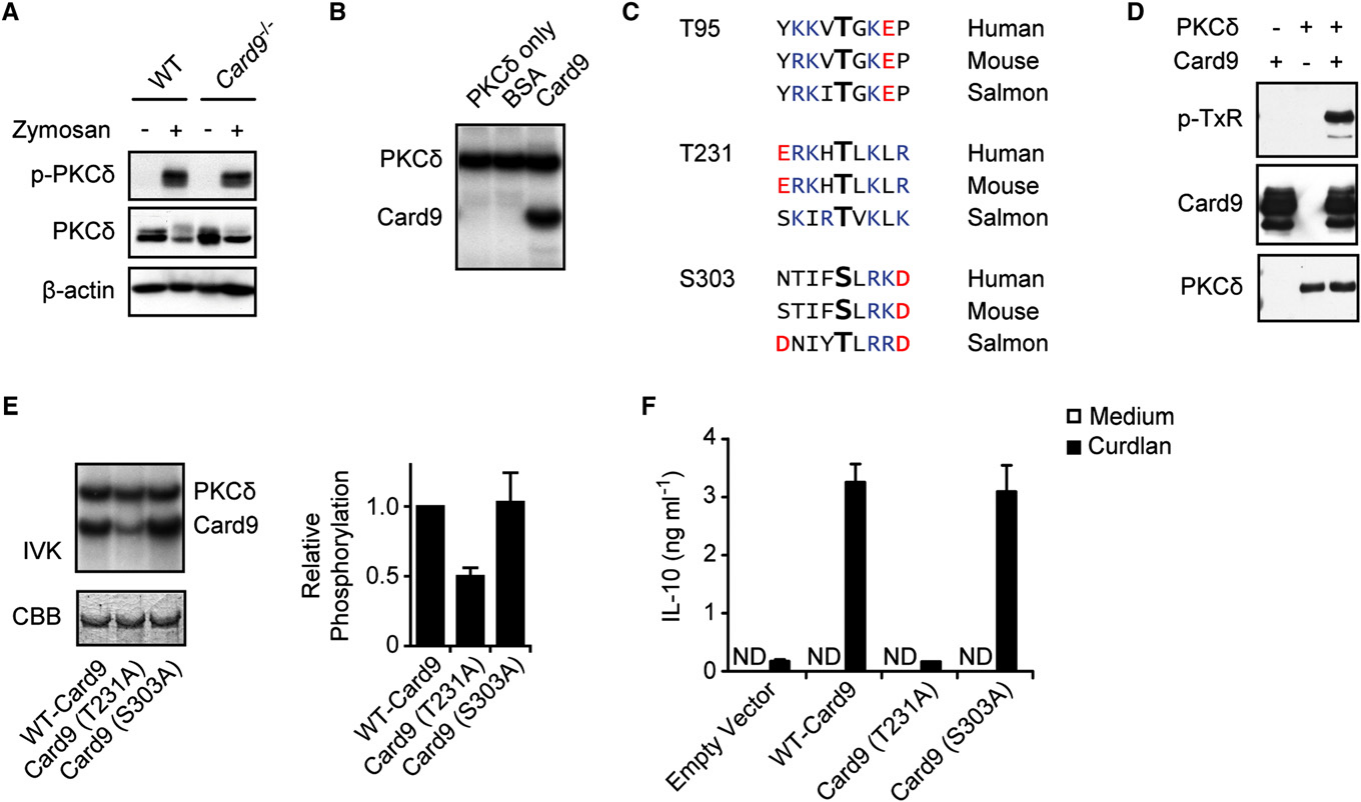
PKCδ Mediates Card9 Phosphorylation for Cytokine Production (A) Normal PKCδ activation in *Card9*^−/−^ cells. BMDCs from WT and *Card9*^−/−^ mice were stimulated for 10 min with zymosan. PKCδ activation was determined by immunoblot with antibodies against phospho-PKCδ, PKCδ, or β-actin. (B) In vitro kinase assay. Recombinant PKCδ was incubated with recombinant Card9 or BSA and γ-[^32^P]ATP for 60 min. Autophosphorylation of PKCδ and phosphorylation of Card9 was visualized by autoradiography. (C) Alignment of highly conserved putative PKC phosphorylation sites within Card9. Basic amino acids are depicted in blue and acidic amino acids in red. (D) Recombinant PKCδ and/or recombinant Card9 was incubated with ATP for 2 hr. Card9 TxR-phosphorylation was determined by immunoblot with antibodies against phospho-TxR, Card9, or PKCδ. (E) Strep-tagged WT-Card9, Card9(T231A), or Card9(S303A) were expressed in HEK293 cells, purified, and incubated in vitro with recombinant PKCδ and γ-[^32^P]ATP. Left: phosphorylation of PKCδ and Card9 mutants were analyzed by autoradiography. Equal Card9 loading was verified by Coomassie Brilliant Blue (CBB) staining. Right: Card9 phosphorylation quantified by densitometry. Results represent means + SD from three independent experiments. (F) Card9-deficient BMDCs were retrovirally reconstituted with empty vector or vectors expressing WT-Card9, Card9(T231A), or Card9(S303A). Reconstituted cells were stimulated with Curdlan and IL-10 concentrations in the supernatants were measured by ELISA. ND: not detectable. Results represent means + SD from one experiment representative of three independent experiments.

As mentioned above, Dectin-1-Syk signaling engages the Card9 adaptor protein for canonical IKK-dependent NF-κB activation (Gross et al., 2006; Mócsai et al., 2010). To study the potential involvement of PKCδ in the Card9 signaling cascade, we first stimulated *Card9*^−/−^ BMDCs (Gross et al., 2006) with zymosan and investigated PKCδ tyrosine phosphorylation. PKCδ was normally activated in the absence of Card9 (Figure 5A). Then, we tested the possibility that PKCδ might directly phosphorylate Card9. Indeed, in vitro kinase assays with recombinant proteins demonstrated that PKCδ can phosphorylate Card9 but not bovine serum albumin (Figure 5B). To search for putative PKCδ phosphorylation sites within Card9, we performed a bioinformatic analysis applying both the NetPhosK (http://www.cbs.dtu.dk/services/NetPhosK/) and Scansite (http://scansite.mit.edu/) target site prediction algorithms (Blom et al., 2004; Obenauer et al., 2003). Three conserved PKC phosphorylation sites were predicted in both murine and human Card9. These were Thr95, Thr231, and Ser303 (Figure 5C). Because Thr95 and Thr231 are within the only two conserved TxK or TxR motifs in Card9, we next performed immunoblot analysis with proteins from the PKCδ-Card9 kinase reaction and a phosphoThreonine-x-Arginine (pTxR) antibody that recognizes phosphorylated Thr followed by Arg or Lys at the +2 position. Consistent with the bioinformatic prediction, PKCδ-phosphorylated Card9 reacted with the pTxR antibody, indicating phosphorylation at TxK motifs (Figure 5D). To further map PKCδ phosphorylation sites within Card9, we used site-directed mutagenesis and transiently transfected expression plasmids encoding tagged forms of WT-Card9, Card9(T95A), Card9(T231A), or Card9(S303A) in HEK293 cells. We were technically unable to efficiently express the Card9(T95A) version in mammalian cells, possibly because of effects of this mutation on protein stability. Nevertheless, WT-Card9, Card9(T231A), and Card9(S303A) were equally expressed in HEK293 cells (data not shown) and were used upon purifications as substrates for in vitro PKCδ kinase assays (Figure 5E). The quantification of radio-phospho incorporation revealed that WT-Card9 and Card9(S303A) were equally phosphorylated by PKCδ in vitro. In contrast, the phosphorylation of the Card9(T231A) mutant was reduced by 50% compared to the WT, demonstrating that PKCδ phosphorylates Card9 at position Thr231 and presumably on additional sites.

To study the functional relevance of the Card9 Thr231 phosphorylation in CLR signaling, we next generated retroviral vectors that express WT-Card9, Card9(T231A), or Card9(S303A) to reconstitute Card9 gene-deficient BMDCs. Subsequently, we stimulated the reconstituted cells with the Dectin-1 agonist Curdlan and measured the production of IL-10 as a readout for productive signaling (Figure 5F). Although the Card9(S303A) mutant restored the signaling defect of Card9-deficient cells to the same extent as WT-Card9, Card9(T231A) was unable to rescue Dectin-1 signaling for cytokine production. Together with the biochemical results above, these data indicate that PKCδ mediates Thr231 phosphorylation of Card9 and that this phosphorylation is essential for Card9 function.

### PKCδ Activates TAK1 via Card9-Bcl10 Complex Formation

**Figure 6:**
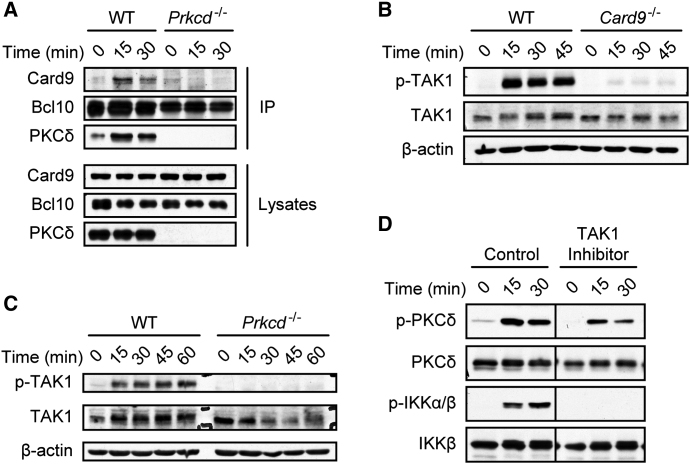
PKCδ Triggers Card9-Dependent TAK1 Activation (A) PKCδ controls Card9-Bcl10 complex assembly. WT and *Prkcd*^−/−^ BMDCs were stimulated with zymosan for the indicated times and lysates subjected to immunoprecipitation with Bcl10-specific antibodies. Immunoprecipitates and total lysates were immunoblotted as indicated. (B) Card9-dependent TAK1 activation. WT or *Card9*^−/−^ cells were stimulated with zymosan as indicated. Lysates were immunoblotted with antibodies against phospho-TAK1, TAK1, or β-actin. (C) PKCδ mediates TAK1 activation. BMDCs from WT or *Prkcd*^−/−^ mice were stimulated as in (B). Lysates were analyzed by immunoblot with antibodies against phospho-TAK1, TAK1, or β-actin. (D) TAK1 signaling is critical for IKK activation. WT BMDCs were pretreated for 30 min with DMSO (control) or the selective TAK1 inhibitor (5Z)-7-Oxozeaenol (2 μM) and stimulated with zymosan. Lysates were analyzed by protein immunoblotting for PKCδ and IKK activation with antibodies against p-PKCδ, PKCδ, p-IKKα/β, and IKKβ.

Upon cellular stimulation, Card9 forms a signaling complex with its effector protein Bcl10 leading to canonical IKK-dependent NF-κB signaling (Gross et al., 2006; Mócsai et al., 2010). To investigate the requirement for PKCδ in these events, we analyzed Card9-Bcl10 complex assembly in *Prkcd*^−/−^ cells. To this end, we immunoprecipitated Bcl10 from zymosan-stimulated WT and *Prkcd*^−/−^ cells and studied its association with Card9 by protein immunoblot. Card9-Bcl10 complexes assembled only in zymosan-stimulated WT BMDCs but not in cells lacking PKCδ (Figure 6A), indicating an essential function for PKCδ activity in Card9-Bcl10 complex formation.

How the Card9-Bcl10 signalosome activates IKKs has not been well defined. However, given that Bcl10 can utilize the kinase TAK1 for T cell receptor-mediated NF-κB activation (Sun et al., 2004), we speculated that TAK1 could also be activated through the Card9-Bcl10 complex during innate immune response. To test this hypothesis, we stimulated BMDCs of the different genotypes with zymosan and investigated TAK1 phosphorylation. Indeed, zymosan-induced TAK1 phosphorylation was strictly dependent on Card9 (Figure 6B). Moreover and in line with our finding that *Prkcd*^−/−^ BMDCs were defective in Card9-Bcl10 activation, zymosan-induced TAK1 activation was abrogated in PKCδ-deficient cells (Figure 6C). To directly investigate the function of TAK1 in zymosan-induced NF-κB signaling, we pretreated BMDCs with the selective chemical TAK1 inhibitor (5Z)-7-Oxozeaenol before cell stimulation (Ninomiya-Tsuji et al., 2003). Although TAK1 inhibition did not block zymosan-induced PKCδ activation, it prevented the activation of IKKs (Figure 6D). Together, these findings indicate that PKCδ plays an essential role upstream of the Card9-Bcl10 module, which is critical for the subsequent activation of TAK1 and the engagement of the canonical NF-κB pathway.

### PKCδ Is Essential for Innate Antifungal Immune Defense

**Figure 7:**
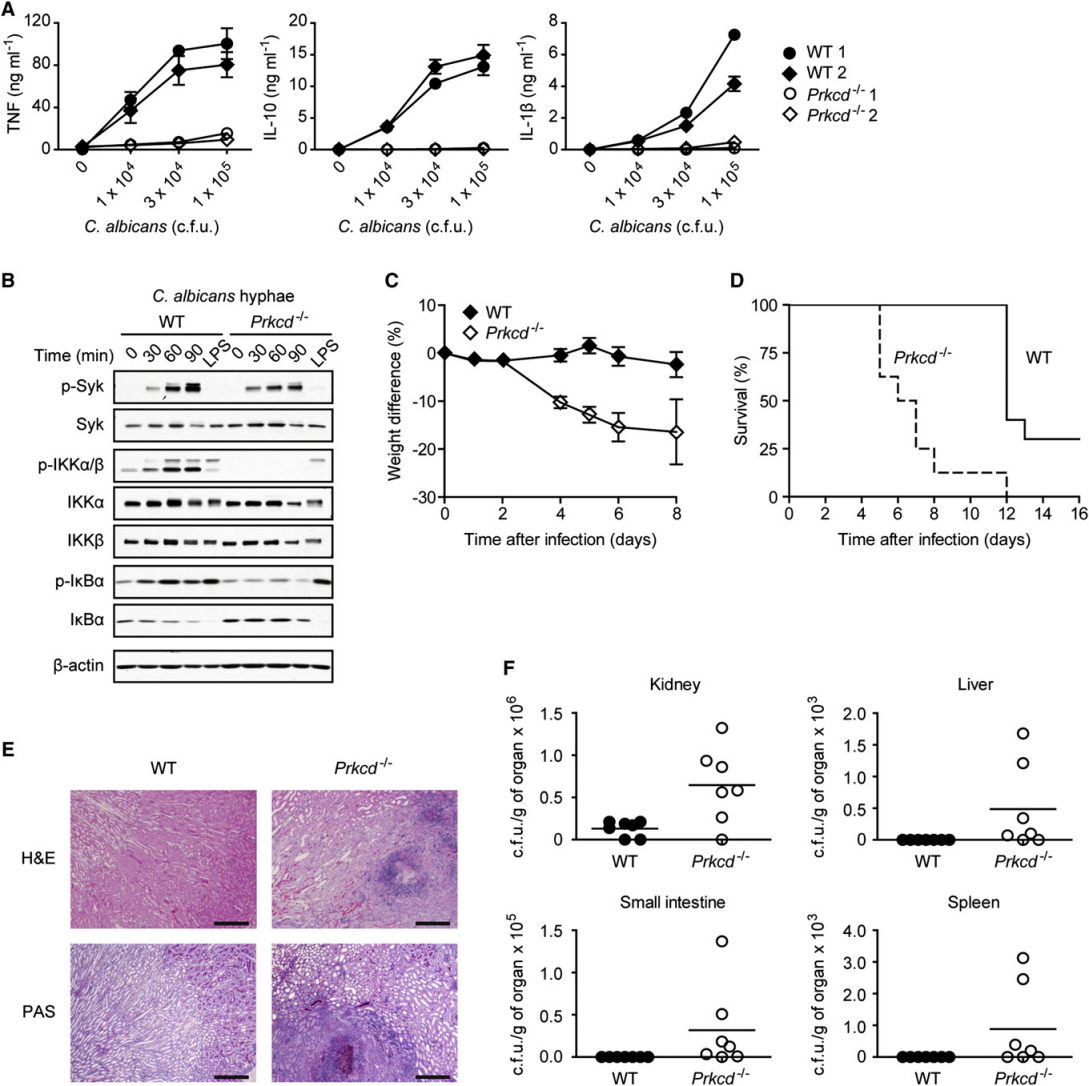
PKCδ Is Critical for Antifungal Host Defense (A) BMDCs from WT and *Prkcd*^−/−^ mice were incubated with increasing doses of live *C. albicans* hyphae. Concentrations of TNF, IL-10, and IL-1β in the culture supernatants were determined 6 hr later. Results are means ± SD of triplicates. (B) BMDCs were stimulated for the indicated times with *C. albicans* hyphae or for 30 min with 100 ng ml^−1^ LPS. Lysates were analyzed by immunoblot with antibodies against phospho-Syk, Syk, phospho-IKKα/β, IKKα, IKKβ, phospho-IκBα, IκBα, or β-actin. (C and D) WT (n = 9) and *Prkcd*^−/−^ (n = 8) mice were infected intravenously with 5 × 10^4^ colony-forming units (c.f.u.) of *C. albicans* and monitored daily for weight changes (C) and survival (D). Statistical analysis was performed by log-rank test (p < 0.0001). (E) Kidney sections from *C. albicans*-infected WT and *Prkcd*^−/−^ mice were stained with H&E or PAS (scale bars represent 300 μm). (F) Mice were infected intravenously with 1 × 10^4^ c.f.u. of *C. albicans*. After 8 days, *C. albicans* titers were determined in the kidneys, the liver, the small intestine, and the spleen.

After uncovering the molecular functions of PKCδ in CLR signaling, we were interested in its role in a pathophysiologically relevant setting. As a clinically important model pathogen we chose *C. albicans* because its PAMPs are recognized by Dectin-1 (Taylor et al., 2007), Dectin-2 (Robinson et al., 2009), and Mincle (Wells et al., 2008) and they drive Syk- and Card9-mediated innate immunity and host defense (Glocker et al., 2009; Gross et al., 2006; LeibundGut-Landmann et al., 2007; Robinson et al., 2009). Although *C. albicans* infection of WT BMDCs induced a robust and dose-dependent production of cytokines, TNF, IL-10, and IL-1β, production of these cytokines was almost completely abolished in the absence of PKCδ (Figure 7A). Also on the biochemical level we observed that, in spite of normal Syk phosphorylation, *Prkcd*^−/−^ BMDCs had severe deficiencies in activating NF-κB signaling after stimulation with *C. albicans* hyphae, a potent agonist of Dectin-2 (Saijo et al., 2010). In line with our results above, *Prkcd*^−/−^ cells furthermore showed defects in IKK activation, as well as in IκBα phosphorylation and degradation (Figure 7B) after *C. albicans* recognition.

Finally, to study the relevance of these findings for host protection in vivo, we infected *Prkcd*^−/−^ mice with *C. albicans*. Compared to the wild-type, PKCδ-deficient mice exhibited significantly greater weight loss upon infection (Figure 7C) and had much lower survival rates (Figure 7D). In an independent set of experiments, we sacrificed the animals 8 days after infection and assessed intravital fungal growth. Consistent with an essential role for PKCδ in innate resistance in vivo, we observed massive fungal infiltration in the kidneys of *Prkcd*^−/−^ mice by histopathology (Figure 7E) and detected significantly higher titers of *C. albicans* in the kidneys, livers, small intestines, and spleens (Figure 7F).

## Discussion

Our results defined an essential role for PKCδ in the activation of CLR-mediated, Card9-dependent innate immune responses. Our findings are in line with the established functions of CLRs and Card9 in antifungal defense (Ferwerda et al., 2009; Glocker et al., 2009; Gross et al., 2006; Saijo et al., 2010; Taylor et al., 2007), and we showed an essential activity of PKCδ in host resistance in vivo.

The detection of fungal particles by Dectin-1 and the subsequent activation of Syk triggers various intracellular signaling pathways (Kerrigan and Brown, 2011; Mócsai et al., 2010; Osorio and Reis e Sousa, 2011). We observed that the recognition of zymosan elicits a Src- and Syk-dependent phosphorylation of PKCδ at Tyr311, indicating that PKCδ activation occurs downstream of Syk. Moreover, *Prkcd*^−/−^ BMDCs showed impaired Card9-Bcl10 complex assembly and NF-κB control in spite of normal Syk activation, demonstrating that PKCδ acts upstream of Card9. Together, these findings indicate that PKCδ operates as a missing link between Syk signaling and Card9 complex formation for the activation of innate immunity. As shown by the fact that only *Prkcd*^−/−^ BMDCs but not cells lacking PKCα, PKCβ, or PKCθ were defective in Dectin-1-induced cytokine production, PKCδ is the specific PKC isoform for signaling in the Dectin-1 pathway.

Treatment of the cells with a small molecule PKC kinase inhibitor blocked zymosan- or curdlan-induced cytokine production. This indicates that the enzymatic serine-threonine kinase activity and not merely a scaffold function of PKCδ is responsible for its activity in Dectin-1 signaling. In vitro kinase assays revealed that Card9 is a direct PKCδ substrate and that PKCδ phosphorylates Card9 at position Thr231. PKCδ is likely to phosphorylate other Card9 residues because the Card9(T231A) mutant was still substantially phosphorylated by PKCδ in vitro. Another PKCδ target site could be Thr95 because global proteomic approaches have demonstrated Card9 phosphorylation at Thr95 in vivo (Choudhary et al., 2009). Because we were unable to express the Card9(T95A) mutant in mammalian cells, we were not able to determine the physiological function of Thr95 phosphorylation but speculate that it might be important for protein stability. Nevertheless, our genetic experiments with Card9-deficient cells that were reconstituted with the phosphorylation-defective Card9(T231A) mutant revealed that the Thr231 residue phosphorylated by PKCδ is absolutely required for downstream signaling and cytokine production. In addition, we observed that PKCδ signaling is essential for Card9-Bcl10 complex assembly and for Card9-dependent TAK1 activation. Thus, we postulate a molecular model in which Syk-induced PKCδ activity mediates direct Card9 phosphorylation, finally resulting in Card9-Bcl10 complex assembly for TAK1 activation. TAK1 then most probably mediates Dectin-1-induced IKK activation in a fashion similar to its mode of triggering NF-κB activity in response to stimuli from other immune receptors, such as TLRs (Vallabhapurapu and Karin, 2009). This would be consistent with the fact that pharmacological TAK1 blockage inhibited zymosan-induced IKK activation.

Intriguingly, although the Card9 signaling pathway was severely defective in *Prkcd*^−/−^ BMDCs, the phagocytosis of zymosan and the production of ROS were largely intact and the activation of Erk MAPK signaling was only slightly reduced. These findings indicate that PKCδ controls only specific subsets of the Dectin-1 responses. Because Dectin-1 ligation can activate the serine-threonine kinase Raf-1 through alternative mechanisms (Gringhuis et al., 2009), it is possible that Raf-1 might be responsible for Dectin-1-triggered and PKCδ-independent Erk activation, consistent with the role of Raf-1 in activating MAPK pathways in numerous settings (Galabova-Kovacs et al., 2006). Interestingly, *Prkcd*^−/−^ mice were similar to *Card9*^−/−^ mice (Gross et al., 2006) in being highly susceptible to fungal infections. The specific PKCδ-Card9 effector response downstream of CLRs is therefore absolutely critical for host defense. The aforementioned pivotal functions of Dectin-1 and Card9 in human antifungal immunity were recently demonstrated in genetic studies (Ferwerda et al., 2009; Glocker et al., 2009). In the future it will thus be important to investigate whether genetic defects in PKCδ might also cause human immunodeficiency syndromes.

Many of our experiments aimed at identifying the principle mechanisms of CLR signaling utilized zymosan stimulation or selective Dectin-1 triggering. Yet in the absence of PKCδ, activation of the NF-κB pathway as well as cytokine production were also severely impaired in response to whole *C. albicans* cells. Recent studies have indicated that *C. albicans* cells, and particularly *C. albicans* hyphae, are potent activators of Dectin-2 signaling (Saijo et al., 2010). Presumably the hyphae in addition also activate Mincle (Wells et al., 2008). Thus, we propose that PKCδ also couples signals from those CLRs to the Card9-controlled NF-κB pathway. This hypothesis is in line with our observation that *Prkcd*^−/−^ BMDCs were defective in cytokine responses to selective agonists for Dectin-2 and Mincle, which formally establishes PKCδ as a general integrator of CLR function.

We believe that our findings have implications beyond antifungal immunity. Mincle and Dectin-1 detect ligands on mycobacteria (Ishikawa et al., 2009; Rothfuchs et al., 2007) and *Card9*^−/−^ mice are impaired in the Mincle-induced inflammatory response to the mycobacterial cord factor (Schoenen et al., 2010; Werninghaus et al., 2009). These animals also succumb rapidly to aerosol lung infection with *Mycobacterium tuberculosis* (Dorhoi et al., 2010). In addition, *Schistosoma mansoni* activates Dectin-2 and Card9 (Ritter et al., 2010) and viruses such as the *Dengue virus* induce inflammatory responses through the ITAM-coupled CLR Clec5a (Chen et al., 2008). Moreover, Dectin-1 also binds to unknown endogenous structures on T cells (Ariizumi et al., 2000), the Syk-coupled CLR Clec9a recognizes ligands that are exposed upon cellular necrosis (Sancho et al., 2009), and Mincle triggers Card9-dependent inflammatory responses upon binding to SAP130 from necrotic cells under sterile conditions (Yamasaki et al., 2008). Together with our results, these data imply that PKCδ might also mediate innate responses to bacteria, parasites, or viruses or could be involved in immune responses to conditions of noninfectious cell injury. These hypotheses need to be investigated.

Finally, CLR-triggered Card9 signaling not only regulates the immediate innate antimicrobial responses but also couples innate recognition to the activation of adaptive immunity (Kerrigan and Brown, 2011; Osorio and Reis e Sousa, 2011). Triggering of the Card9 pathway by Dectin-1, Dectin-2, or Mincle ligands on antigen-presenting cells controls the synthesis of distinct cytokines. This cytokine milieu instructs the development of antigen-specific Th17 cell responses and additionally induces Th1 cell immunity (LeibundGut-Landmann et al., 2007; Robinson et al., 2009; Saijo et al., 2010; Werninghaus et al., 2009). Th17 cell responses are often associated with autoimmunity and polymorphisms in Card9 are recurrently detected in human inflammatory diseases (Pointon et al., 2010; Zhernakova et al., 2008). It will therefore be important to test whether PKCδ signaling in innate cells couples to Th17 cell responses and whether aberrant activity in CLR-induced PKCδ-Card9 signaling contributes to human inflammatory disease. In this context, PKCδ is a drugable target and PKC inhibitors are in clinical trials. Thus, our findings raise the possibility of therapeutically manipulating CLR-mediated Card9 signaling by targeting PKCδ function.

## Experimental Procedures

### Mice and *C. albicans* Infections

*Prkca*^−/−^, *Prkcb*^−/−^, *Prkcq*^−/−^, *Prkcd*^−/−^, and *Card9*^−/−^ mice have been described (Gross et al., 2006; Leitges et al., 1996, 2001, 2002; Pfeifhofer et al., 2003). Mice were infected with 1 × 10^4^–5 × 10^4^ colony-forming units (c.f.u.) of *C. albicans* (strain SC5314) as described (Gross et al., 2006) and monitored daily for health and survival, according to institutional guidelines. For fungal burden determination, the organ homogenates were plated in dilutions on Sabouraud agar. For histological analyses, kidney sections were stained with hematoxylin and eosin or periodic acid-Schiff according to standard protocols.

### BMDC Culture and Cell Stimulation

BMDCs were derived from bone marrow as described (Gross et al., 2006) and stimulated with zymosan (Sigma), curdlan (Wako), LPS, CpG, Pam_3_CSK_4_, poly(I:C) (all from Invivogen), Dectin-2 monoclonal antibody (AbD Serotec), TDB (Avanti Polar Lipids), or *C. albicans* strain SC5314, as indicated. Where indicated, cells were pretreated for 20 min with the Syk inhibitor R406 (Rigel), the Src kinase inhibitor PP2 or its inactive analog PP3 (both Calbiochem), the PKC inhibitor panPKC LMWI (ALTANA Pharma, Konstanz, Germany), or the TAK1 inhibitor (5Z)-7-Oxozeaenol (Calbiochem Merck).

### Purification and Identification of Tyrosine-Phosphorylated Proteins

2 × 10^8^ BMDCs were stimulated for 10 min with zymosan (300 μg ml^−1^) and lysed in RIPA buffer. The lysates were then subjected to immunopurification with 20 μg of either an antibody against phospho-tyrosine (P-Tyr-100) or mouse IgG1 as an isotype control coupled to Protein A Sepharose. Immunopurified proteins were eluted with Phenylphosphate (100 mM) in PBS, concentrated by ultrafiltration, and separated by 1-D PAGE. Whole gel lanes were cut into 23 slices. After protein digestion, MS-MS spectra of individual gel slices were obtained with a liquid chromatography coupled LTQ-Orbitrap XL hybrid mass spectrometer (Thermo Electron). Peak lists were searched against NCBInr database with Mascot v.2.2.06 as a search engine and MudPIT for protein scoring.

### In Vitro Kinase Assays

Recombinant PKCδ was purchased from Proqinase (Heidelberg) and in vitro kinase assays were performed according to manufacturers' instructions. Recombinant Card9 as a substrate was purified from *E. coli* by standard protocols. Alternatively, the indicated Card9 mutants containing a Strep-tag were transiently transfected into HEK293 cells and were purified with Streptactin-sepharose (IBA BioTAGnology). In brief, 70 ng PKCδ were incubated with 2 μg BSA, 2 μg Card9 (*E. coli*), or 1 μg HEK293 expressed Card9-versions in 70 mM HEPES-NaOH (pH 7.5); 3 mM MgCl_2_; 3 mM MnCl_2_; 3 μM Na_3_VO_4_; 1.2 mM DTT; 50 μg ml^−1^ PEG20.000; 100 nM ATP; 5 μCi γ-[^32^P]ATP; and 1% (v/v) DMSO in 20 μl for the indicated times. Samples were boiled in Laemmli buffer, separated by SDS-PAGE, and analyzed by autoradiography. For quantification, densitometric phospho-band signals were normalized to Coomassie Brilliant Blue stain intensities.

### Retroviral Reconstitution of Card9-Deficient BMDCs

Retroviral particles were generated by transfection of Phoenix-E cells with pMIG retroviral vectors expressing the indicated mutants of human Card9, as previously described (Glocker et al., 2009). Bone marrow cells of *Card9*^−/−^ mice were retrovirally transduced on day 1 and 2 of BMDC differentiation. Successful transduction was verified by FACS analysis of GFP expression from the bicistronic pMIG retroviral vectors and protein immunoblot analysis of Card9 expression, as previously described (Glocker et al., 2009).

### Cytokine Production

Concentrations of TNF, IL-10, IL-2, and IL-1β secreted into cell culture supernatants of untreated and stimulated cells were analyzed with plate-bound ELISA-kits (ELISA Ready-SET-Go!, eBioscience or BD OptEIA, BD Biosciences PharMingen) according to manufacturers' recommendations.

### Zymosan Phagocytosis

BMDCs were left untreated or incubated for 30, 120, or 180 min with FITC-labeled zymosan particles (100 μg ml^−1^, Molecular Probes). After washing, cells were analyzed either by microscopy for FITC fluorescence or by flow cytometry for CD11c expression and FITC fluorescence in the CD11c-positive population with a FACSCanto II (BD Biosciences) and FlowJo Software (Tree Star, Inc.) according to standard protocols. Fluorescently labeled antibodies were from eBioscience.

### Reactive Oxygen Species Measurements

2 × 10^5^ BMDCs were plated in 100 μl culture medium without GM-CSF into a 96-well luminometer plate (Nunc). For stimulation, 100 μl HBSS containing 100 μM Luminol and zymosan was added to each well. The luminensence was measured for 1 hr in 10 min intervals at a Mithras LB940 according to manufacturers' instructions. As a measure for ROS generation, relative light units (RLU) were integrated over time.

### Signal Transduction

Protein immunoblots were performed as described (Gross et al., 2006) with primary antibodies against Syk, phospho-Syk (Tyr525/526), PLCγ2, phospho-PLCγ2 (Tyr759), phospho-Tyrosine (P-Tyr-100), PKCδ, phospho-PKCδ (Tyr311), phospho-IKKα/β (Ser176/180), p44/42 MAPK (Erk1/2), phospho-p44/42 MAPK (Erk1/2) (Thr202/Tyr204), IκBα, phospho-IκBα (Ser32/36), phospho-TxR, TAK1, phospho-TAK1 (Thr184/187), Bcl10 (all Cell Signaling Technology), IKKβ, IKKα (Upstate Millipore), Card9 (Santa Cruz Biotechnology), or β-actin (Sigma). For coimmunoprecipitation experiments, BMDCs were stimulated with zymosan as indicated and lysed in NP-40-containing buffer. Lysates were cleared by incubation with Protein G Sepharose (GE Healthcare 4 Fast Flow) and then incubated with a Bcl10 antibody. Immune complexes were precipitated with Protein G Sepharose and subjected to protein immunoblotting as indicated.
